# Pore-scale physics of ice melting within unconsolidated porous media revealed by non-destructive magnetic resonance characterization

**DOI:** 10.1038/s41598-024-56294-w

**Published:** 2024-03-07

**Authors:** Natnael Haile, Muhammad Sajjad, Yadong Zhang, Nahla AlAmoodi, Faisal AlMarzooqi, TieJun Zhang

**Affiliations:** 1 Department of Mechanical and Nuclear Engineering, Khalifa University of Science and Technology, P.O. Box 127788, Abu Dhabi, United Arab Emirates; 2 Department of Chemical and Petroleum Engineering, Khalifa University of Science and Technology, P.O. Box 127788, Abu Dhabi, United Arab Emirates

**Keywords:** Mechanical engineering, Hydrology

## Abstract

Melting of ice in porous media widely exists in energy and environment applications as well as extraterrestrial water resource utilization. In order to characterize the ice-water phase transition within complicated opaque porous media, we employ the nuclear magnetic resonance (NMR) and imaging (MRI) approaches. Transient distributions of transverse relaxation time *T*_2_ from NMR enable us to reveal the substantial role of inherent throat and pore confinements in ice melting among porous media. More importantly, the increase in minimum *T*_2_ provides new findings on how the confinement between ice crystal and particle surface evolves inside the pore. For porous media with negligible gravity effect, both the changes in NMR-determined melting rate and our theoretical analysis of melting front confirm that conduction is the dominant heat transfer mode. The evolution of mushy melting front and 3D spatial distribution of water content are directly visualized by a stack of temporal cross-section images from MRI, in consistency with the corresponding NMR results. For heterogeneous porous media like lunar regolith simulant, the *T*_2_ distribution shows two distinct pore size distributions with different pore-scale melting dynamics, and its maximum *T*_2_ keeps increasing till the end of melting process instead of reaching steady in homogeneous porous media.

## Introduction

Melting of ice at pore-scale has a large variety of applications including thawing of permafrost in mountains and polar regions, food and cold energy storage^1,2^. For space exploration, harvesting water from ice deposits in Moon (with ~330 billion kilograms of water ice^3^) and Mars regolith can significantly reduce the cost of space missions^4–6^. In order to harvest these water resources, systematic studies are required to understand the pore-scale phase transition, particularly the melting dynamics of ice crystals among regolith particles. Existing investigations on ice-water phase change, predominately at bulk scale, consider the effects of porosity and the relative impact of conduction and natural convection as well as the thermal conductivity of porous matrices^7–10^. However, the interfacial physics of ice-melting at pore-scale is not well understood, particularly for heterogeneous porous media. At the interfacial level, when two particles are in proximity in the presence of wetting fluid, a capillary bridge (i.e., liquid-particle interface) is formed between them, causing particles to adhere to each other^11^. The resulting interactions (e.g. capillary forces) depends on the physical properties of the fluid-particle system and operating conditions^12^. Interaction forces between particles are also affected by the phase transition of the trapped fluid (e.g., water to ice)^13^. It is feasible to characterize interactions between two particles with trapped liquid/ice through colloidal probe atomic force microscopy^14^ and surface force apparatus^15^, however, liquid-particle system becomes complicated when particles are packed together to form porous media. Therefore, it is challenging to study interfacial interactions and their impact on phase transitions, such as pore-scale ice-melting, due to the opaque nature of porous media. Moreover, the liquid/ice-particle system becomes more complicated in porous media composed of heterogeneous particles like Moon regolith. For example, Weaver and Viskanta^7^ conducted an experimental and analytical study to evaluate if free convection is a major heat transfer mode during melting of frozen porous samples. The melting experiments were performed in a small horizontal and vertical cylindrical bottles filled with different small-sized spherical soda-lime glass and aluminum beads as porous matrix, where water was used as the phase change material. The bead size affects the melting rate, where the melting process is fastest for the large-size bead and slowest for the small glass bead in the vertical orientation. As for the porous sample made from aluminum beads, conduction was significant because of the higher thermal conductivity of porous matrix^8^. Beckermann and Viskanta^9^ also conducted experimental and numerical study of the ice-water phase change in porous media using a volume-averaged transport equation. The experiments were carried out in a vertical square-shaped enclosure with large sized glass beads (6 mm in diameter) and gallium acting as porous matrix and fluid, respectively. They found that the conduction in the ice region and free convection in the melt region have high impact on the shape and motion of melting front.

The advent of technologically modern tools such as nuclear magnetic resonance (NMR) and magnetic resonance imaging (MRI) makes it possible to study the melting dynamics without destroying opaque materials’ structure or disturbing phase transition processes. The NMR spectroscopy offers insights into the system's structure and dynamics through chemical shift measurements and analysis of the corresponding relaxation properties^16^. Nuclear magnetic resonance cryoporometry (NMRC) is also one of the methods employed for measuring pore size distributions of mesoporous material (2 nm–1 μm)^17,18^, especially during phase change process^19–21^. These solid–liquid phase transitions (i.e., melting and freezing) can be construed to obtain pore size distribution and shape of the porous media^22^. NMRC is based on the Gibbs–Thomson equation, where the melting point depression of a liquid confined within a pore is inversely related to the pore size^19^. Therefore, the Gibbs–Thomson transformation is employed to convert melting-point depressions into pore sizes, while the Strange-Rahman-Smith transformation is applied to determine the porosity at various pore sizes through differentiation and re-mapping of the melting curve data^23^. For example, Sarah et al.^19^ conducted ^1^H NMRC experiments on the melting of aqueous NaCl solution in porous materials, and they successfully converted NMRC signal intensities into pore size distributions by the standard Gibbs–Thomson analysis. Similarly, ^1^H NMRC has been employed to investigate nitrobenzene's freezing and melting dynamics in mesoporous silicon with ink bottle geometry, which possesses different pore sizes and pore structures^20^. The study found that materials with pores of uniform channel-like features exhibit specific freezing and melting transitions that depend on the size of the pores. Furthermore, in smaller channels, the freezing process shows the dominant impact of the pore-blocking mechanism, which delays the propagation of a solid front by the channel necks^20,24^. Freezing and melting experiments of nitrobenzene confined within pores of Vycor porous glass and random pore structure are also carried out using NMRC, where different solid–liquid configuration within the porous sample is found due to different cooling and warming histories (i.e., hysteresis)^25^. The pore-blocking causes freezing transition through invasion percolation, while melting occurs uniformly throughout the pore network.

NMR relaxometry (time-domain NMR) can be utilized to study transport properties of porous materials with pore size in the range of micrometers^26,27^. The relaxometry signal amplitudes are directly proportional to the liquid content of porous sample’s^28^. Hence, they can be used in measuring water content as a function of time. The NMR relaxation times can be longitudinal (*T*_*1*_) or transversal (*T*_*2*_), which depends on the surface-to-volume ratio of a pore. The NMR relaxometry has been used to calibrate water retention curves from NMR experiments conducted on partially saturated soils^29,30^, which provides insights into transport processes in porous media and helps in understanding the intertwined physics of capillary pressure and liquid saturation^31^. NMR *T*_*2*_ signal for solids is generally in μs while for liquids is in ms. The substantial difference in* T*_*2*_ signal for solids and liquid enables identification of the different phases (e.g., water and ice) inside the porous media^17^. The objective of this work is to perform in-situ characterization of water–ice phase change in homogeneous and heterogeneous porous media by employing NMR relaxometry. In porous media, there is no sharp ice melting front, instead a mushy zone exists around the melting front. It remains challenging to characterize the evolution of mushy frontier and associated ice melting dynamics in opaque porous media. In fact, the melting front is controlled by the ice melting dynamics at pore scale because the ice crystals formed inside the porous matrix introduce confinements in addition to pores and throats. In this study, by looking into the varying distributions of NMR transversal relaxation time *T*_*2*_, we reveal how the crystal-induced confinements evolve during ice melting and how they shape the phase transition at the pore scale. Bulk-scale mushy ice melting front is also studied with cross-sectional images of spatial water distribution from MRI. Moreover, the temperature distributions as a function of time and position are captured through thermocouple measurements for porous media under both vertical and horizontal orientations to explore the impact of the transport properties of porous media on melting dynamics. Heat transfer analysis of the moving water–ice interfaces is performed to reveal the energy transport mechanism and provide more insights into the melting process. Our findings at pore and bulk scales also elucidate the role of capillary and gravity forces that drive different ice melting dynamics in various porous media.

## Materials and methods

### Materials

**Figure 1:**
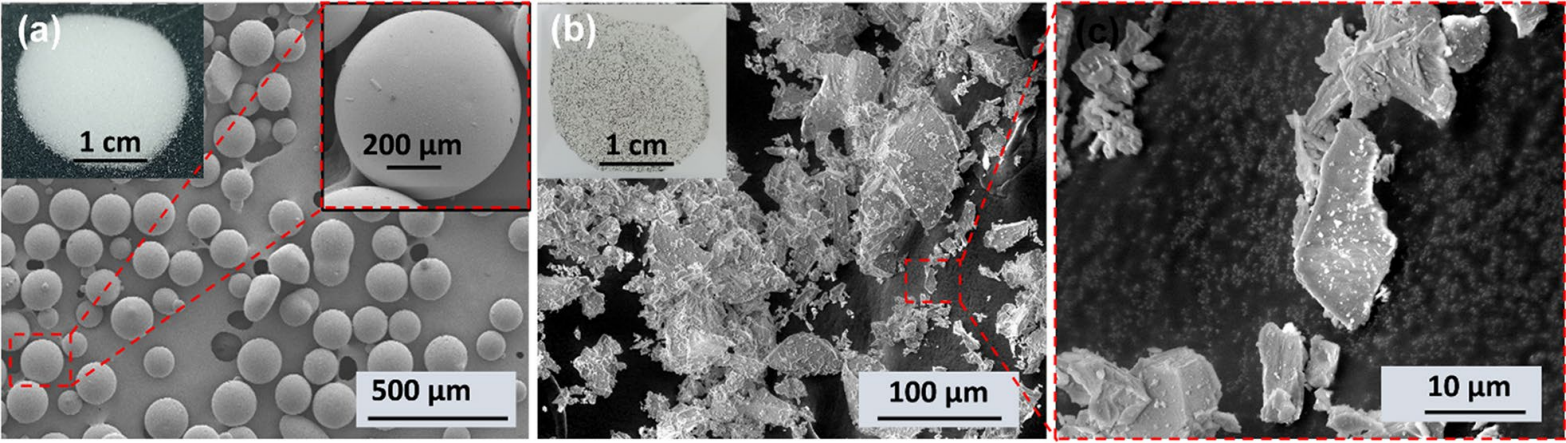
Low and high magnification Scanning Electron Microscope (SEM) images of (**a**) homogenous silica glass beads, and (**b**) heterogenous lunar highlands simulant. The inset optical images in (**a**,**b**) represent white-colored glass beads and gray-colored lunar highlands simulant, respectively.

In this study, both homogeneous and heterogeneous porous materials are considered to evaluate the ice melting process. For controlled experiments, homogeneous porous structures are made of spherical soda lime (silica) glass beads with a range of diameters (1–50, 70–110, 100–200 µm). Silica is the most abundant mineral on earth, so the silica glass beads are used as the soil replica with similar thermophysical properties to study the associated water–ice phase change processes^32^. These silica glass beads are intrinsically hydrophilic with microscopic contact angle of around 20°^33,34^. Heterogeneous media in this study is made of regolith simulant, which is a synthesized terrestrial material that resembles the chemical, mechanical, mineralogy, and particle distribution of other planetary regolith such as the moon and Mars. There are two types of lunar regolith: the highlands and mare simulant, where the highlands refer to the light region of the moon, and mare refers to the dark region. One of the highlands simulants is the lunar highlands simulant (LHS), which is a mineral-based, high-fidelity simulant that adequately simulates regolith for an average or general highlands location of the moon. This simulant is made to resemble the texture of lunar regolith by coupling both mineral and rock fragments in precise proportions instead of a single terrestrial lithology, and it is mainly composed of silica (51.2%), alumina (26.6%) and calcium oxide (12.8%)^35^. The surface morphology of the employed glass beads and LHS is characterized using Scanning Electron Microscope (SEM) as shown in Fig. 1. The glass beads are in spherical shape with almost negligible macro-scale roughness as shown in Fig. 1a. However, the shape of LHS particles is highly irregular and rock fragments in the sample can be easily observed from low and high magnification SEM images given in Fig. 1b. For sample preparation, the beads or LHS were inserted in a small cylindrical bottle with a height of 40 mm and a diameter of 24 mm separately. The trapped air in the porous media was removed via vacuum. Then, deionized (DI) water was siphoned into sample, followed by 1.5-h ultrasonication to homogenize the distribution of glass beads. After that, the water-saturated sample is kept for around 24 h at a freezer temperature of – 20 °C. Finally, the frozen sample is inserted inside the NMR detection coil for the melting experiment. In order to study the melting process in vertically orientated porous samples, the details on the employed thermocouple-embedded experimental setup are provided in Section S1 of the Supplementary Information.

### Transversal relaxation

In the non-destructive NMR characterization, transversal relaxation measurement is critical to evaluate the porosity, pore-size distribution, permeability, and other properties of opaque porous media^36^. Prior knowledge about the NMR relaxation of water (proton) is a prerequisite to understand the application of NMR in melting dynamics of ice water in porous media. The transverse magnetization vector dephasing of the precessing protons is the cause for the transversal relaxation. Such relaxation happens only due to dephasing between the spinning nuclei without any transfer of energy to the surrounding; hence it is called spin–spin relaxation. Therefore, the transversal relaxation time $\left( {T_{2} } \right)$, the time constant of the transverse magnetization decay, is represented by^37,38^:

1 $$\frac{1}{{T_{2} }} = \frac{1}{{T_{{2,{\text{ bulk}}}} }} + \frac{1}{{T_{{2,{\text{ surface}}}} }} + \frac{1}{{T_{{2,{\text{ diffusion}}}} }}$$

where the bulk relaxation (*T*_*2, bulk*_) of a fluid is the property affected by the chemical composition as well as viscosity. The presence of gradient in a magnetic field and longer inter-echo spacing results in diffusion-induced relaxation (*T*_*2, diffusion*_), which is mostly initiated when molecules move to a place where the magnetic field strength differs. Inhomogeneity in the static magnetic field across different locations causes extra dephasing. Surface relaxation (*T*_*2, surface*_) takes place at the boundary between the fluid and the neighboring solid interface. In porous materials, the existing surface of the porous matrix (i.e., glass beads or regolith) is the most suitable for such a type of relaxation.

In a wetting fluid such as water, molecular motion is mostly restricted at the interface between the porous material surface and fluids. Such restriction causes differences in the diffusion coefficient when a fluid is inside porous media or in its bulk form, provided both are held at the same pressure and temperature^37^. However, the diffusion-induced relaxation becomes negligible when the inter-echo spacing is kept low (≅ 0.2 ms). In addition, the surface relaxation time of water is much smaller than the bulk relaxation time. Water molecules at the surface have strong interaction with pore wall and minimal interaction with similar molecules, unlike those in the bulk. The relaxation rate of proton at the pore surface depends on its frequency of collision with the pore wall. The smaller the pore size, the higher the relaxation rate (or shorter the surface relaxation time). However, the molecules away from the pore wall have smaller relaxation rate (or larger bulk relaxation time, *T*_2,bulk_) as compared to those near the pore wall, and hence the term 1/ *T*_2,bulk_ in Eq. (1) is often neglected. As a result, the transversal relaxation time of water can be simplified to^37,39^:

2 $$\frac{1}{{T_{2} }} = \rho_{2} \left( \frac{S}{V} \right)$$

where $\rho_{2}$ represent the surface relaxivity and (*S/V*) denotes the surface-to-volume ratio of a given pore body. The surface relaxivity values for porous media with glass beads sizes of 50 μm, 100 μm and 200 μm are 14.9 μm/s, 16.5 μm/s and 21.8 μm/s, respectively^40^. For regular pore shapes such as sphere with radius *r*, the (*S/V*) ratio is equal to 3/*r*^37^. In porous media saturated with fluid, the surface-to-volume ratio is inversely proportional to pore size distribution, such that for small-sized pores, the ratio is large and vice versa. Therefore, the *T*_2_ spectrum is simply another illustration of the pore size distribution with larger *T*_2_ values representing bigger pore sizes as indicated by Eq. (2). The variation in surface area-to-volume ratio is expressed by the shift of *T*_2_ spectrum, from which the water distribution and transport inside the porous medium can be determined. $T_{2}$ distribution is obtained from the raw magnetization decay (echo train) through an inversion method, so called echo-fit or mapping, and the mathematical procedure is known as inverse Laplace transformation. After inverting the raw NMR data, the transversal relaxation curve (*T*_2_) is obtained. Then, from the *T*_2_ distribution, the *T*_2, *min*_ and *T*_2, *max*_ are obtained which are related to the smallest and largest pore sizes, respectively. The shift in *T*_2, *min*_, *T*_2, *max*_ and  ${\Delta }T_{2} = T_{2, max} - T_{2, min}$ is monitored to study the melting dynamics in the porous samples.

### NMR-integrated experimental setup and calibration

**Figure 2:**
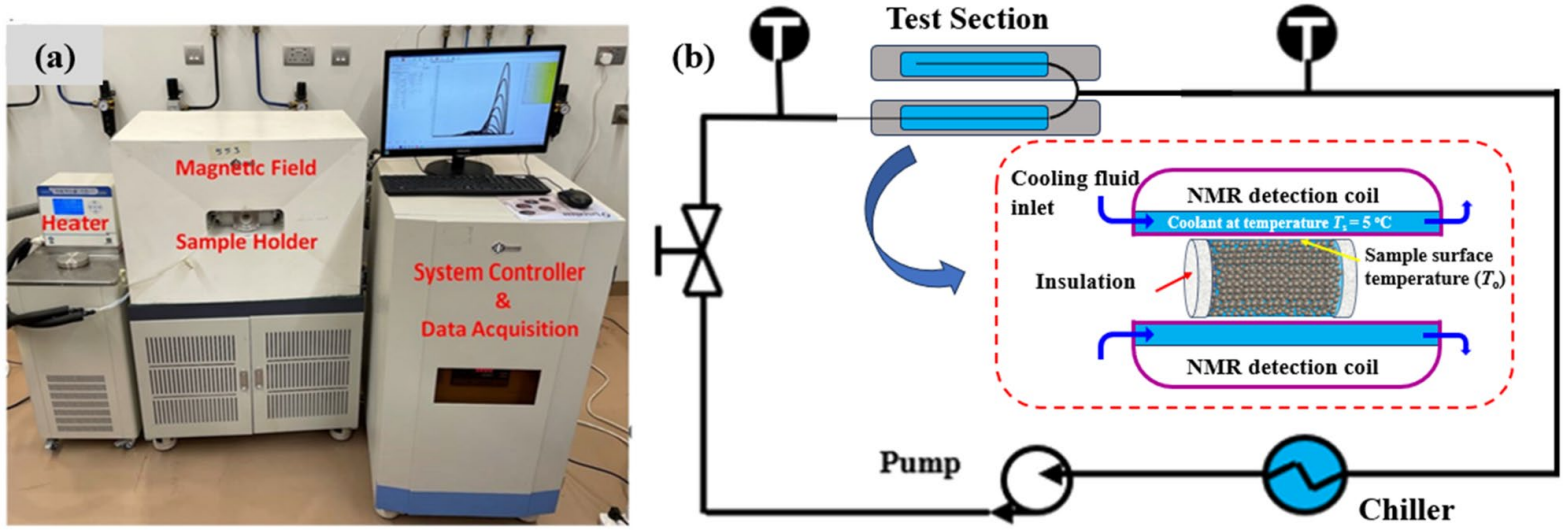
(**a**) A photograph of the NMR-integrated experimental setup; (**b**) Schematic of NMR-integrated flow loop. Porous sample is located at the center of NMR detection coil in the horizontal orientation, while its sidewall temperature is maintained by circulating coolant flow. Inset in (**b**) shows the detailed schematic of the NMR test section with porous sample.

NMR experiments are conducted through a low-field nuclear magnetic resonance analyzer (Niumag, MesoMR23-060H-I) with a magnetic field strength of 0.5 Tesla (Fig. 2). The experimental ice melting setup is embedded in the NMR–MRI characterization system, as shown in Fig. 2a. The magnetic pole generates the static magnetic field, and its operating temperature is held constant at 32 °C. The frozen porous sample is placed horizontally inside the detection coil of the NMR analyzer. The front and back surfaces of the sample holder are insulated, while the lateral surface temperature is maintained at 5 °C by a temperature control bath. Similarly, the porous sample is insulated from the front and back. As a result, melting starts from the edge of the lateral wall and progresses radially. In order to control the melting processes, a cold fluid is supplied around the cylindrical bottle to cool the sidewall of the sample (Fig. 2b). A cooling fluid (Fluorine oil FC-40) is used that does not have any hydrogen nucleus. As a result, the NMR signals are not affected by the cooling oil. In addition, a low-humidity environment is highly desirable during low-temperature NMR experiments to avoid air condensation. For this purpose, the front and back openings of the detection coil were sealed carefully.

Porous samples are located at the center (sweet spot) of the radiofrequency (RF) coil, where the magnetic field is homogenous. The RF coil applies an oscillating magnetic field and causes the rotation of net magnetization from the longitudinal to transverse plane. When the melting process proceeds, the receiver coil records the sample’s magnetization decay, which is then converted to data that relate the transverse relaxation time and amplitude of the magnetization decay. The resonance frequency (*f*_r_) of RF coil depends on the inductance (*L*) and capacitance (*C*) of the resonance loop as: $f_{r} = 1/\left( {2{\uppi }\sqrt {CL} } \right)$^41^. For this NMR analyzer, the RF frequency is adjusted to match the central frequency of protons, which is the sum of frequency factory setting (SF = 21 MHz) and frequency offset (O1). A standard sample composed of *D*_2_O and *H*_2_O is usually employed while using free induction decay (FID) sequence. The quality factor (Q) can be related to the *f*_r_ and its bandwidth (Δ* f*_r_) as: Q = *f*_r_/ Δ* f*_r_^42^. The sharper the amplitude vs frequency curve, the higher the Q factor and signal to noise ratio (SN)^43^. However, high Q factor also increases the response time (τ) of the receiving coils^44^, which reduces its sensitivity. Another way to improve SN of the NMR–MRI system is to increase the number of scans (*N*_s_) as SN ∝ *N*_s_^½^^41^. In this study, we set the number of scans to 16 for high value of SN.

The measurement was conducted by the CPMG pulse sequence after its inventors Carr, Purcell, Meiboom and Gill, in which a 90° pulse is followed by consecutive 180° pulses^45^. The NMR analyzer scans every 4 min. Before performing experiments, the NMR signal of the dry porous medium (zero saturation) is captured as a baseline and subtracted from the total signal of the water-saturated sample during melting. As a result, the *T*_2_ distribution is solely based on the water siphoned to the porous matrix. To obtain the transient water content during the melting process, calibration is carried out first by using the standard sample. Based on standard NMR samples, a linear relationship between the mass of water (*m*_w_) and area under *T*_2_ distribution (*A*) is observed as: *m*_w_ = *a A*. Since *m*_w_ is known for standard samples and corresponding *A* is obtained from *T*_2_ distributions, slope *a* can be easily determined from standard samples and can be used for actual samples to determine amount of water. After getting the transient mass of water, the melting rate can be determined by subtracting the mass of water content at two consecutive times and dividing it by the time interval. In addition, MRI images were also captured to illustrate the spatial distribution of water during the transient melting process. The MRI spectroscopic frequency is 21 MHz. The images show the evolution of the melting front, which is helpful to study the dominant heat transfer mode. In addition, the MRI can only detect a given substance in its liquid state because the solid ice has an ultrafast relaxation time that is characterized by a noisy signal. As a result, the MRI image displays a white color for liquid water while both ice and solid matrix are dark.

## Results

The melting dynamics of ice in porous media characterized by non-destructive NMR results in a set of *T*_*2*_ distributions. The melting rate, *T*_*2, min*_ and *T*_*2, max*_ curves can be obtained from these distributions to evaluate transient melting behaviors of homogeneous and heterogeneous porous media.

### Transient characterization of ice melting in homogeneous porous media

#### NMR characterization of ice melting among glass beads

**Figure 3:**
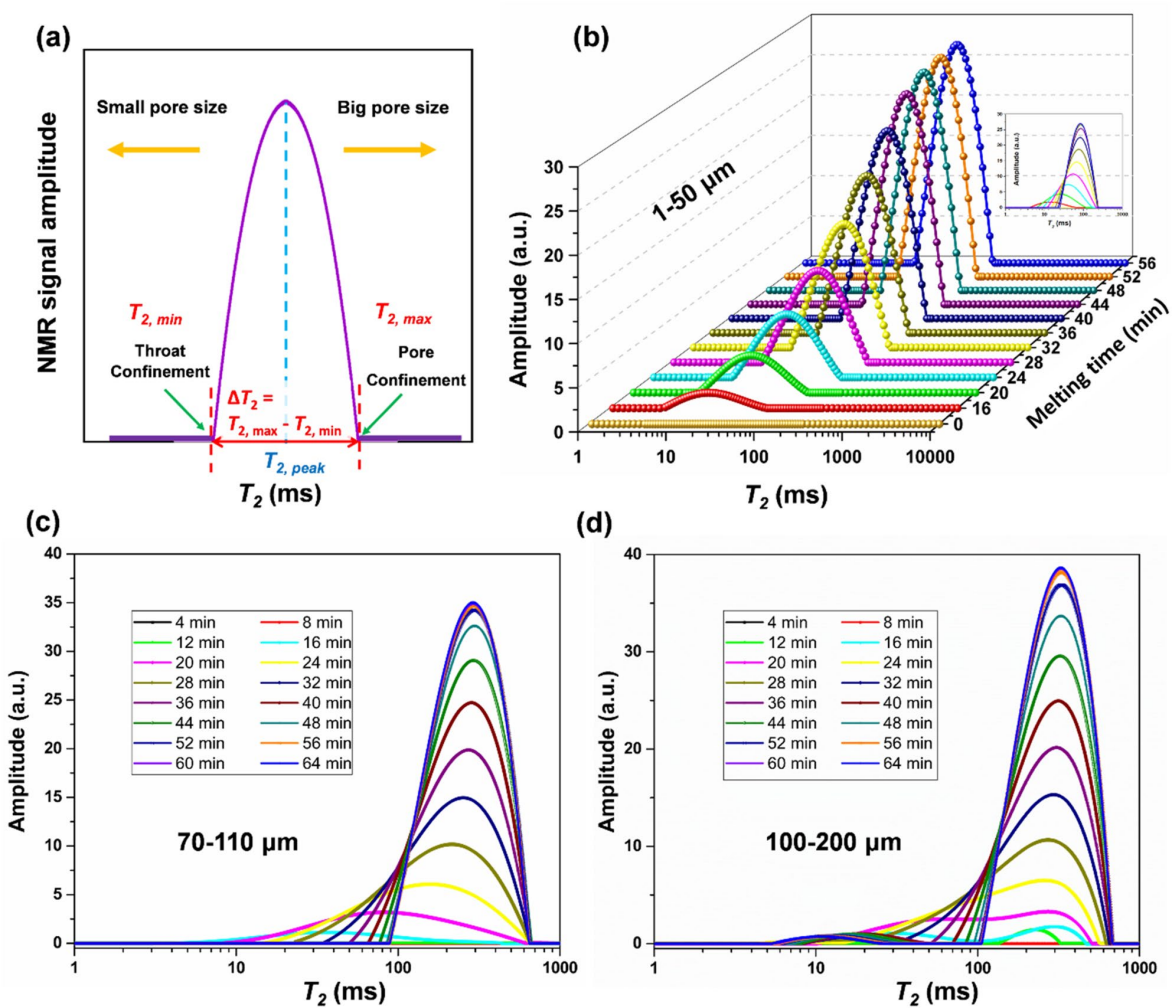
(**a**) Typical *T*_2_ distribution of homogeneous porous media. *T*_2_ distributions of ice melting in porous media with glass beads size (**b**) 1–50 µm, (**c**) 70–110 µm, and (**d**) 100–200 µm.

Figure 3a shows typical *T*_2_ distribution for homogeneous porous media. *T*_2, *min*_ and *T*_2, *max*_ are related to the smallest and largest pore sizes, respectively as shown in Fig. 3a. Similarly, *T*_2, *peak*_ represents the time at which the signal amplitude reaches its maximum value as indicated in Fig. 3a. The *T*_*2*_ distribution of ice melting in porous glass beads media with size 1–50 µm, 70–110 µm and 100–200 µm are shown in Fig. 3b–d. For porous media with 1–50 µm glass beads, it took ~56 min for melting to complete, as seen in the Fig. 3b. The *T*_*2*_ distribution shows an increase in amplitude, indicating that the water content is increasing with time. Similarly, for glass beads with size range of 70–110 µm and 100–200 µm (Fig. 3c,d), it took ~64 min to complete the melting process. As surface area to volume ratio (*S*/*V*) is inversely proportional to particle diameter, porous media with 1–50 μm glass beads will have higher surface area as compared to the one composed of 100–200 μm glass beads. This will result in slightly higher melting rate of ice within the tight porous media, as compared to the coarse porous media.

**Figure 4:**
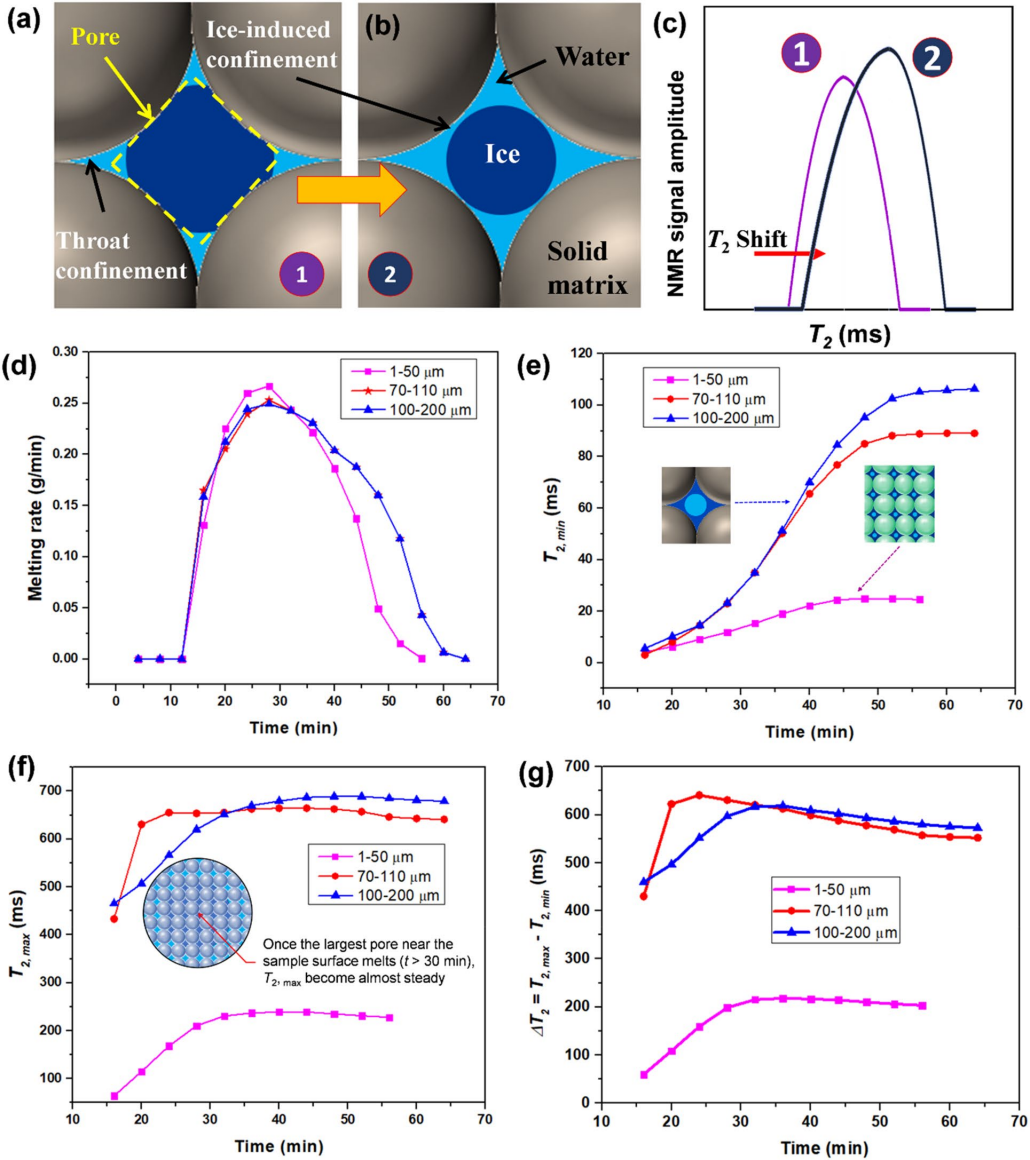
(**a**–**c**) Illustration for melting of ice inside a pore with a corresponding shift in *T*_2_ distributions. Inherent pore/throat confinements and ice-induced confinements are also demonstrated in (**a**,**b**). (**d**) Transient ice melting rate; (**e**–**g**) Evolution of *T*_*2, min*_, *T*_*2, max*_ and Δ*T*_2_ = *T*_2, *max*_ − *T*_2, *min*_ during ice melting process, respectively.

The melting of ice inside a pore is illustrated in Fig. 4a,b with corresponding *T*_2_ distributions demonstrated in Fig. 4c. There are three types of confinements: inherent pore and throat confinements; and ice-induced confinements (Fig. 4a,b). The shift in *T*_2_ distributions towards the right in Fig. 3a–d (as illustrated in Fig. 4c) indicates that the size of the ice-induced confinements increases as the ice melts inside the pores. For instance, the size of the ice-induced confinement in Fig. 4b is higher than that of Fig. 4a owing to the melting of ice crystal inside the pore. The area under the* T*_*2*_ distribution represents the amount of ice melted. The melting rate in Fig. 4d is calculated from the NMR data by subtracting the peak area magnitude (the amount of melted water) between consecutive scans and then the difference divided by the time span (4 min). For porous samples with a size range of 1–50 µm, the melting rate curve shows zero value during the first 12 min as sample temperature was below bulk melting point. This could happen because the sample was kept in the freezer at a temperature of − 20 °C. At the 12th minute, the melting rate starts to increase until it reaches a peak value (0.27 g/min) and then decreases towards zero. This pattern is observed in all the porous media samples. It is happening partly because of the difference in thermal conductivity of the ice, water, glass beads and the cylinder wall, which results in variation of the effective thermal conductivity with temperature. The thermal conductivity of the glass bottle wall (~1 W/m K), soda lime glass beads (~0.937 W/m K) and ice (~2.2 W/m K) is much higher than water (~0.591 W/m K)^9^. Therefore, the effective thermal conductivity of ice-glass beads is higher than water-filled glass beads. In particular, the dominance of ice would speed up the melting process until it reaches a peak melting rate close to 0.27 g/min. However, at the late stage, when the content of water after melting overtakes the solid ice, the effective thermal conductivity experiences a reduction and accordingly, the melting rate declines. More importantly, the increase in temperature of porous sample during the melting process reduces the thermal gradient between the sample surface and surrounding, resulting in a gradual reduction in melting rate in the late stage. A detailed heat transfer analysis based on radial heat conduction is provided in Section S2 of the Supplementary Information. Similarly, the melting rate trend of the remaining two experiments is the same as the previous one, except that the peak melting rate is slightly different. For a porous samples with 70–110 µm and 100–200 µm glass beads, the peak melting rates are 0.25 and 0.24 g/min, respectively (Fig. 4d).

Figure 4e shows the variation of *T*_*2, min*_ as a function of melting time for glass bead sizes of 1–50 µm, 70–110 µm and 100–200 µm, respectively. All three *T*_*2*_ distributions start with almost the same *T*_*2, min*_ value, which indicates the dominance of ice-induced confinements (between ice crystal and particle surface inside the pore) in the early stage of melting process for all three types of porous media_._ As melting proceeds, the ice-induced confinement in Fig. 4a becomes larger, and it shifts *T*_*2, min*_ towards the right. Compared to porous media with large-sized glass beads, the inherent pore confinement among small-sized glass beads will be much higher than the ice-induced confinements as illustrated in Fig. 4e. In coarse porous media with large glass beads, the ice-induced confinement plays a more dominant role than the inherent pore confinement, leading to a significant increase in *T*_2, min_. This implies more obvious shift in *T*_2, min_ towards the right for 100–200 µm in comparison with 1–50 µm as confirmed by our results in Fig. 4e. At the end of ice melting process, no more ice-induced confinements exist and *T*_2, min_ represents size of the throat confinement. Note that the throat confinement in coarse porous media (with 100–200 µm glass beads) is ~ 4 times of that in tight porous media (with 1–50 µm glass beads). *T*_2, max_ (Fig. 4f) represents the largest pore size, and its shift towards longer relaxation time indicates the weak confinement after ice melting. For instance, ice-induced pore confinement illustrated in Fig. 4b would vanish and accordingly, a big pore (near the surface of the porous sample) would appear once all ice inside that pore is melted. This also indicates that large confinements will dominate over the smaller ones during the melting process as confirmed by the shift in *T*_2, peak_ towards the right in Fig. 3b–d. *T*_2, max_ at the end of the melting process will become higher for porous media with large-sized glass beads compared to the one with small-sized glass beads, as shown by the results given in Fig. 4f. To compare the shift in *T*_2, max_ and *T*_2, min_ towards longer relaxation time, their difference (i.e., Δ*T*_2_ = *T*_2, *max*_ − *T*_2, *min*_) is plotted in Fig. 4g. Δ*T*_2_, representing broadness of the *T*_2_ peak, initially increases with melting time and reaches a peak value as shown in Fig. 4g. However, a slight reduction in Δ*T*_2_ happens at the end of melting process owing to increasing *T*_2, *min*_ and almost steady *T*_2, *max*_ as shown in Fig. 4e,f.

#### MRI imaging of melting front movement in mushy zone

**Figure 5:**
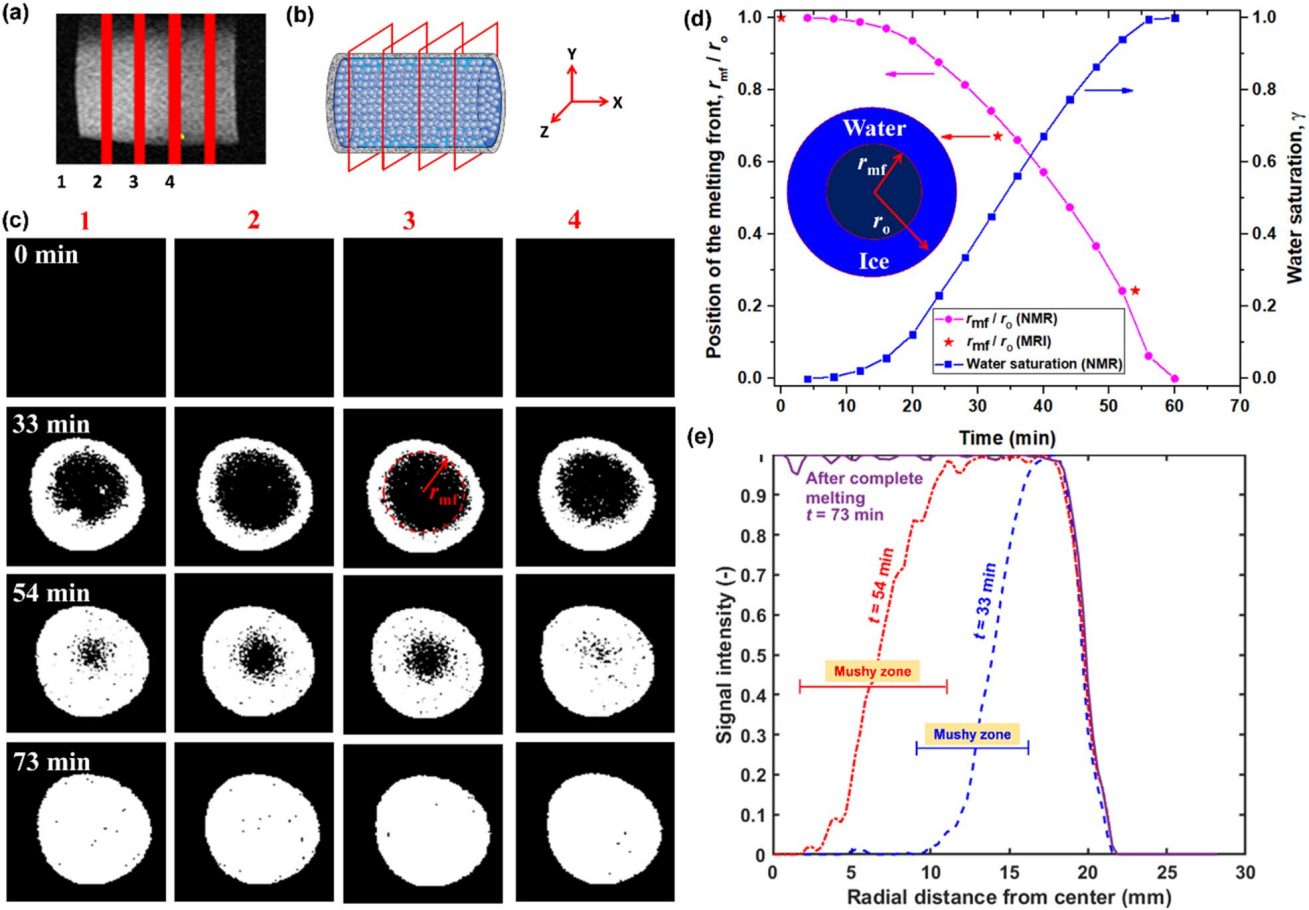
(**a**) Locations/slices for MRI imaging with (**b**) 3D schematic illustrating the position and plane of slices. Slice 1 is located closer to the front section of the sample. (**c**) MRI images during the ice melting process for glass beads with a size of 100–200 μm. (**d**) Variation in dimensionless radial position of the melting front (*r*_mf_/*r*_o_) and water saturation as a function of melting time based on MRI and NMR data for glass beads with size of 100–200 μm. An average value for* r*_mf_/*r*_o_ is taken for MRI images at different cross-sections. (**e**) Normalized signal intensity as a function of radial distance from the sample centre at *t* = 33 min, *t* = 54 min and *t* = 73 min (i.e., after complete ice melting) for slice 2.

In-situ characterization of mushy zone front during ice melting is challenging for opaque porous media. Fortunately, MRI imaging along with NMR transverse relaxation *T*_2_ distribution can be employed to explore the spatio-temporal variation in melting front of the mushy zone. The MRI images of icing porous media were taken in the Y–Z coordinate (cross-section plane) with four slices at different locations of the porous sample, as shown in Fig. 5a,b. The MRI images were captured during the melting process to observe the evolution of the phase change frontier (i.e., melting front) as shown in Fig. 5c. The width of each slice is around 5 mm. The first image was dark in color, indicating that melting had not started. However, at $t$ = 33 min, the white color appears around the rim of the dark circle, which means the ice melts radially at all the axial locations. In the following images, the white circle continues to grow, with the melting front (solid–liquid interface) moving toward the center radially. Along with the radial movement of the melting front (Fig. 5), more pores are full of water as the result of melted ice, which is consistent with the larger magnitude of *T*_2,peak_ and the shift in *T*_2,peak_ towards longer transverse relaxation time (Fig. 3b). Our results given in Fig. S2 of the Supplementary Information show that the amplitude of the *T*_2_ distributions gradually increases with the movement of the melting front towards the center. Also, the radial movement of the melting front with such a regular shape is strong evidence of conduction being the existing heat transfer mode in the horizontal orientation for all bead size ranges. The contribution of the convection heat transfer is not seen even when the melting front is close to the center. This could be related to the diminished effect of the permeability, which is dominant in the vertical orientation. It is also pertinent to mention that the phase change frontier is not sharp and a mushy zone exists between solid and liquid zones as shown in MRI images in Fig. 5c. The evolution of the mushy zone is a common characteristic of phase change materials during melting process^46^. The ice-induced and inherent pore and throat confinements predominately evolve in the mushy zone, and the corresponding NMR results with detailed analysis have been discussed in previous section. The radial position of the melting front from the center of the porous media (*r*_mf_) normalized with maximum radius (*r*_o_) obtained from MRI and NMR is plotted in Fig. 5d for porous media with a glass bead size of 100–200 μm. The* r*_mf_/*r*_o_ can be obtained by processing MRI images in Fig. 5c. The water saturation or liquid fraction (γ) can be related to the position of the melting front (*r*_mf_) and sample radius (*r*_o_) as: γ = 1-(*r*_mf_/*r*_o_)^2^. Thus, *r*_mf_ can be calculated with γ, which is obtained from the NMR *T*_2_ distribution, as shown in Fig. 5d. A good agreement between NMR and MRI results for *r*_mf_/*r*_o_ exists. The s-shaped water saturation curve follows the melting rate profile given in Fig. 4d. Moreover, based on MRI images given in Fig. 5c, the results for radially averaged signal intensity as a function of sample radius are shown in Fig. 5e (Fig. S3 in Supplementary Information). The signal intensity is one for the melted region while it is zero in the region with ice. The higher the spread of the signal, the wider the mushy zone. It can be seen that the mushy zone at *t* = 54 min is wider than that of at *t* = 33 min, demonstrating the growth of the mushy zone along with melting.

### Transient NMR characterization of ice melting in heterogeneous regolith simulant

In the above section, physical insights into the melting dynamics of ice in homogeneous porous media (glass beads) were obtained through experimental and analytical studies. Here we apply the same characterization procedures to heterogeneous porous media. In particular, lunar highlands simulant is studied with more focus as it reflects the soil type of the southern pole of the moon, which is believed to have ice-water deposits^35,47^. The particle size range of the lunar highland’s simulant is very wide (1–1000 µm)^48^, which means the porous sample contains a combination of extremely fine grains and relatively large grains.

**Figure 6:**
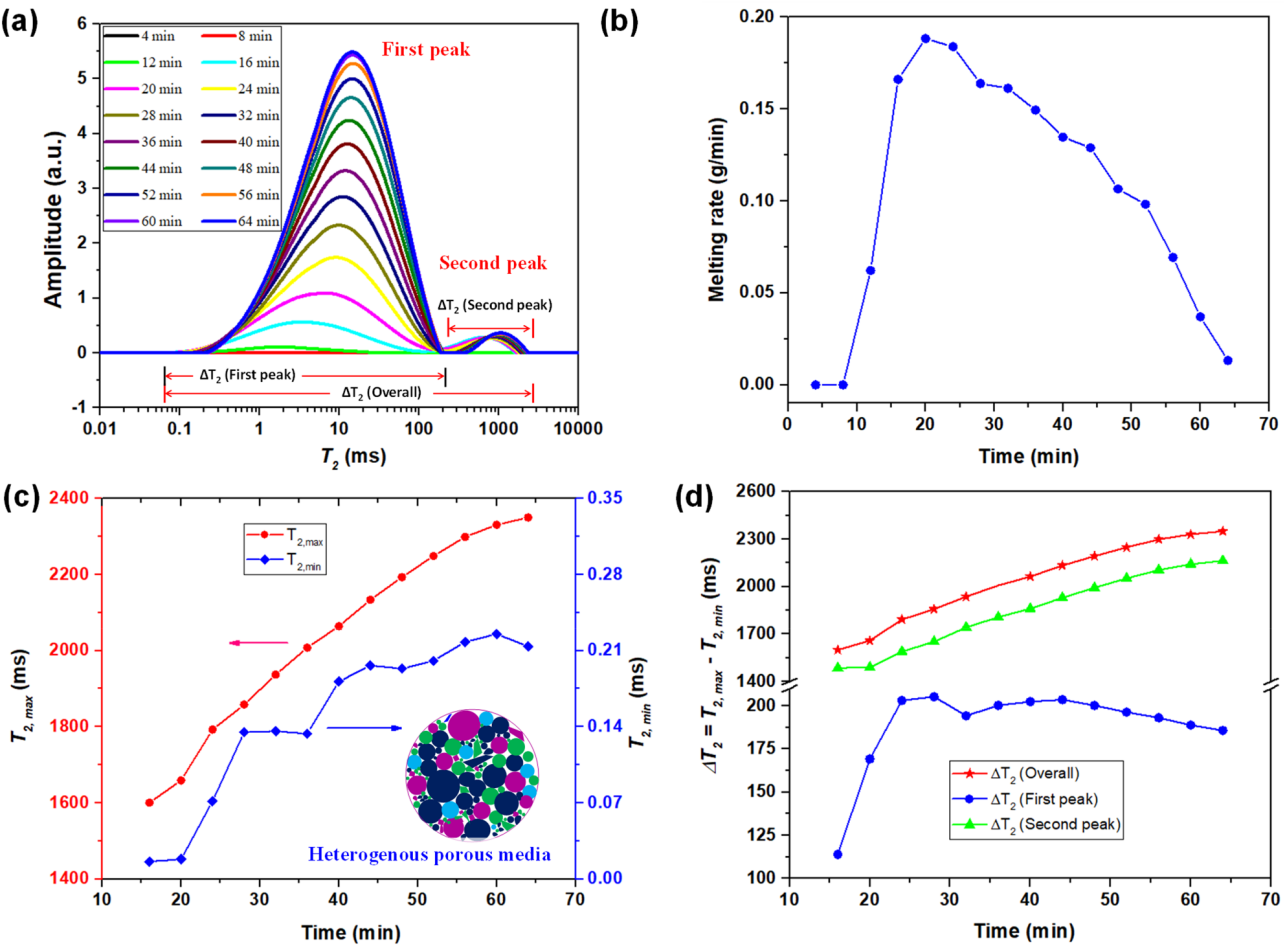
Ice melting in heterogeneous porous regolith (i.e., lunar highlands simulant): (**a**) Evolution of $T_{2}$ curves; (**b**) ice melting rate in g/min; Variation in (**c**) $T_{2, peak}$ and $T_{2, min}$ and (**d**) Δ*T*_2_ for two peaks respectively.

The evolution of transverse relaxation time of ice melting in LHS porous media is shown in Fig. 6a. From the $T_{2}$ curve, the melting process took ~64 min to complete, which is similar to the melting time of the glass beads with the size range of 70–110 µm and 100–200 µm. Unlike the $T_{2}$ curve of the soda lime glass beads, here the curve shows two peaks and indicates the high degree of heterogeneity in the grain size distribution, consistent with the particle size distribution (0.01–1000 µm)^48^. The left $T_{2, peak}$ shifted towards longer time, indicating the initialization of the melting process in the smaller pores of regolith simulant before proceeding to the larger pores (Fig. 6a). As *T*_2, peak_ for the second peak is smaller than the first peak, there is less water in large pores in comparison with smaller pores. The melting rate curve for the LHS in Fig. 6b shows similar patterns as the glass beads, exhibiting a subcooled state at the beginning and then reaching a peak melting rate (~0.19 g/min). The peak value for melting rate occurs at ~20 min, which is lower than that of homogenous porous media (i.e., ~28 min) (Fig. 4d). In comparison with silica-based homogenous porous media (Fig. 4d), the early-stage sharp increase in melting rate for heterogeneous LHS is owing to the higher thermal conductivity of Al_2_O_3_ (~30 W/m K), and CaO (~19.5 W/m K) because these two elements contribute to 40% of the total composition. Also in LHS, the pore and throat could be very small due to irregular shape and broad size distribution of particles (0.01–1000 μm), which results in tight porous media with rapid ice melting, similar to the case of tight homogenous porous media in Fig. 4d. The increase in sample surface temperature results in a reduction in melting rate after the peak value. A shift in $T_{2, min}$ and $T_{2,max}$ towards the larger relaxation time can be seen from Fig. 6c. $T_{2,min}$ shift towards right follows the same trend as in the case of homogenous porous media (Fig. 4e), though heterogeneous LHS exhibits very small $T_{2,min}$ owing to tiny pore and throat size as demonstrated in Fig. 6c. Also, several fluctuations are observed in the $T_{2,min}$ (Fig. 6c) mainly due to the heterogeneity in the size distribution. However, $T_{2,max}$ is kept increasing during the whole melting process for regolith simulant unlike homogenous porous media, where it achieves almost steady value after 34 min (Fig. 4f). For homogenous porous media (Fig. 3a), once ice in the largest pore near the sample surface melts (t > 30 min), *T*_2_, _max_ becomes steady as shown in Fig. 4f while the *T*_2, peak_ curves shift towards right. This means that the number of the largest pores with melted ice (water) increases as the melting front moves radially inward (Fig. 5). However, for regolith simulant, *T*_2, max_ shifts to a larger relaxation time during the whole melting period, which implies that melting of ice in the largest pore gradually takes place and lasts till the end of melting process (Fig. 6a,c). This is attributed to the slow melting of ice in big pores compared to that of ice in small pores. The increasing $T_{2,max}$ for regolith simulant also results in the broadness of the overall pore distributions as indicated by increasing overall Δ*T*_2_*.* The Δ*T*_2_ corresponding to the first peak reduces and the peak becomes narrow after 24 min of melting, a trend similar to homogenous porous media (Fig. 4g). However, the second peak gradually becomes wider mainly owing to the progressive increase in $T_{2,max}$ (Fig. 4g).

## Discussion

The degree of confinement profoundly impacts the physiochemical nature of water and renders property deviation from its bulk phase^49^. The interaction between the pore surface and the water molecule plays a crucial role in altering the H-bond networking, while most of the water molecules close to the pore surface have one free O–H group (CN < 3), resulting in H-bond network discontinuity. This effect becomes more significant as the surface area of interaction increases relative to the bulk volume^50^. The confinement impacts the thermodynamic state of the substance inside the pores and causes changes in the phase diagram, including a shift in the triple point as it depends on pore size^51,52^. For hydrophilic surfaces, there will be stronger molecular interaction between water and the pore wall, while the opposite happens in the case of hydrophobic surfaces^53^. As a result, the partial ordering and interaction of the water molecules with the hydrophilic surfaces lowers the chemical potential of water, causing the depression of melting point ($\Delta T_{m}$)^54^ as expressed by Gibbs–Thomson Eq. ^55–57^:

3 $$\Delta T_{m} = \frac{{V_{m} \gamma_{sl} T_{m} }}{{\Delta H_{f} }}\left( \frac{S}{V} \right)$$

where $T_{m}$, $\gamma_{sl}$, *V*_m_, $S/V$, and $\Delta H_{f}$ represent melting point of bulk water, interfacial tensions between solid–liquid interface, molar volume of water, surface area to volume ratio of pore (Eq. 2) and latent heat of melting, respectively. For ice-water systems at ambient conditions, $T_{m}$ is 273.15 K, $\gamma_{sl}$ is 0.0267 J/m^2^, $\rho_{l}$ is 997 kg/m^3^ and $\Delta H_{f}$ is 333.5 kJ/kg. The pore size and $\Delta T_{m}$ have an inverse relation, that is, the depression decreases with an increase in pore size. Based on NMR data for *T*_2, min_ and *T*_2, max_, $\Delta T_{m}$ of water confined in pore and throat of porous media is estimated as given in Table S1 of the Supplementary Information. The $\Delta T_{m}$ for throat confinement is higher than pore confinement for all samples. The $\Delta T_{m}$ for pore confinement for 1–50 µm, 70–110 µm and 100–200 µm samples are found to be 0.006 °C, 0.002 °C and 0.001 °C, respectively. Similarly, the $\Delta T_{m}$ for throat confinement are 0.06 °C, 0.02 °C and 0.01 °C for 1–50 µm, 70–110 µm and 100–200 µm samples, respectively. The $\Delta T_{m}$ for heterogeneous LHS is expected to be more significant as reflected by *T*_2_ distribution curves in Fig. 6. Note that in the above calculation, the enthalpy of fusion is taken by considering the ice in its bulk form, while confinement has a remarkable impact in reducing the enthalpy of fusion though no reliable analytical relation is available.

The results given in Fig. S4a,b, indicate that melting time increases with the increase in pore size for horizontally oriented samples. However, an opposite trend is observed for vertically orientated samples as increase in pore size reduces melting time, in consistency with the earlier investigations^7,8^. As shown in Table S2 and Fig. S4b of Supplementary Information, the results under vertical orientation indicate faster ice melting in large glass beads as compared to small glass beads. This means that the melting front was moving slightly faster inside large pores than small pores, which shows that natural convection is the major heat transfer mode in the vertically oriented porous media. Along with the melting ice within the top part of the sample, the density increases because of water density inversion behavior, and gravity drives water to the bottom section to displace the colder ice. This happens at a relatively fast rate among the large-sized pores, because water has lower flow resistance in larger pores and natural convection enhances heat transfer besides conduction. As a measure of the fluid flow velocity across porous media, permeability has been recognized as the major factor that causes faster melting among large glass beads in vertical orientation^58^. The detailed transient permeability characterization of porous samples is given in Section S4 of Supplementary Information. The permeability is proportional to the pore size (Fig. S4c,d of Supplementary Information). The measured permeability is 15.98 Darcy for glass beads with a size range of 100–200 µm. However, for the tight porous media with the smallest bead size (1–50 µm), the permeability reaches a value of 0.41 Darcy, which shows the strong resistance of porous media for fluid flow. Therefore, in porous media with the large bead size (100–200 µm), natural convection dominates owing to higher permeability (i.e., 15.98 Darcy), while conduction is the main heat transfer mode for porous media with the smallest bead size (1–50 µm) and low permeability (i.e., 0.41 Darcy). Temperature distribution measurements were also collected for icing regolith simulant for comparison with soda lime glass beads results. As indicated in Table S2 of Supplementary Information, the time it takes for melting to proceed for the lunar highlands simulant is close to the melting time of the soda lime glass beads (100–200 µm). This agrees with the comparison made on the NMR results of the lunar highlands simulant and soda lime glass beads.

It should be noted that all of the experiments conducted on the regolith simulants are taken in our lab by considering the ambient pressure, temperature and gravity. On Earth, the atmospheric pressure is 101,325 Pa (~ 1 bar), while on the moon the pressure drops to $3 \times 10^{ - 15}$ bar^59^, and the gravity is 1/6 of that on Earth. Despite the difference, surface tension plays a dominant role in tight porous media over other physical parameters since the bond number (*B*_o_) of lunar highlands simulant reaches an extremely small value $(B_{o}\ll1)$. In addition, for such microsized porous samples, the pore diameter is extremely small, which reinforces the capillary effect. Therefore, the impact of experimental condition difference between the moon and Earth is limited, particularly for porous media under horizontal orientation.

## Conclusions

As an attempt to probe the physics of ice-water interfacial evolution inside the pores of homogeneous and heterogeneous porous media, experiments have been systematically conducted by applying both the NMR–MRI characterization and temperature distribution measurements. The impact of interfacial and gravitational forces on ice melting and water transport has also been studied with the permeability characterization of porous media. Based on the melting physics of ice in various homogenous porous media, containing silica colloids/beads of different sizes, we have revealed the impact of pore-scale ice-water interface evolution on bulk-scale melting rate and movement of the melting front.

Our NMR–MRI characterization results show that the ice melting rate in tight porous media is slightly higher than that in the coarse one, and the melting rate gradually increases to a peak value and then decreases to zero at the late stage. Our findings indicate that in coarse porous media (with 100–200 μm glass beads/colloids), ice-induced confinement plays a more important role in affecting ice melting dynamics than the throat-imposed confinement. The resemblance of the ice-water interface evolution at the pore-scale, represented by the *T*_2, min_ curve in Fig. 4e, with the bulk-scale water saturation curve in Fig. 5d shows that the pore-scale interfacial phase change dominates the overall melting process. Through direct MRI characterization, we have obtained the temporal cross-section images of bulk-scale melting front with a pore-scale mushy zone, which comprises of ice-induced confinements. Based on the MRI images of regular-shaped melting front movement, we confirm that heat conduction is the major heat transfer mode among all horizontally oriented porous media, in consistency with our theoretical analysis for phase change heat transfer. Contrary to the melting dynamics of porous samples placed in the vertical orientation, ice within the smaller glass beads (with low permeability) melted somewhat faster than in the larger ones in the horizontal orientation owing to the dominance of conduction over convection. Unlike silica glass beads, the *T*_2_ distribution for lunar highlands simulant showed two peaks and indicated a high degree of heterogeneity in the grain size distribution. The melting time of 64 min for LHS was similar to that of the porous sample with 100–200 μm sized glass beads but with a slightly lower melting rate of 0.19 g/min. For heterogeneous LHS, *T*_2_, _max_ keeps increasing till the end of melting process in contrast to that for homogeneous porous media, indicating big pores filled with water emerge at the end of the melting process. These findings on ice melting in homogeneous and heterogeneous porous media have contributed to in-depth understanding of ice-water-particle interaction at pore scale. They are not only valuable to energy storage applications and environmental conservation on the earth, but also instrumental to rational design and technology development for in-situ resource utilization from moon and Mars regolith towards deep space exploration.

### Supplementary Information

Supplementary Information.

## Data Availability

The data used to support the findings of this research are included within the paper.
