# Supplementary material for: Pore-scale physics of ice melting within unconsolidated porous media revealed by non-destructive magnetic resonance characterization
Source: Sci Rep. 2024 Mar 7;14:5635. doi: 10.1038/s41598-024-56294-w (PMC10920668; doi:10.1038/s41598-024-56294-w)
Supplement: Supplementary file 1 — Supplementary Information. [file 41598_2024_56294_MOESM1_ESM.pdf]

Supplementary Information

Pore-scale Physics of Ice Melting within Unconsolidated Porous Media revealed by non-destructive Magnetic Resonance Characterization

Natnael Haile[a,1], Muhammad Sajjad[a,1], Yadong Zhang[a], Nahla AlAmoodi[b] , Faisal AlMarzooqi[b], and TieJun Zhang[a,*]

[a]*Department of Mechanical and Nuclear Engineering, Khalifa University of Science and Technology, Abu Dhabi, P.O. Box, 127788, United Arab Emirates*

[b]*Department of Chemical and Petroleum Engineering, Khalifa University of Science and Technology, Abu Dhabi, P.O. Box, 127788, United Arab Emirates*

[1]*Equivalent first authors;* [*]*Correspondence Email: tiejun.zhang@ku.ac.ae.*

S1 Thermocouple-embedded Experimental Setup

In the NMR analyzer, samples are restricted to be placed in horizontal orientation and effect of gravity on ice melting cannot be evaluated. Hence, we have also conducted temperature distribution experiments in vertical orientation by installing thermocouples at different locations in the porous sample (Fig. S1) since these measurements are complementary to the NMR characterization of ice melting dynamics. In this way, we are also able to capture the temperature distribution over time at different locations and determine the ice melting rates in porous media with different-sized glass beads. As shown in Fig. S1, the vertically oriented cylindrical sample holder contains the glass beads, with thermocouples inserted through sideway slots. Since the diameter of thermocouples is 1mm, they have good contact with glass beads and negligible effect on the melting process. Each melting experiment ran for two hours, and the temperature data were recorded every second throughout the experimental period. The cylinder is insulated from all sides except the top, which is attached to a cold metal plate cooled with a chiller-controlled temperature at 5 ℃.

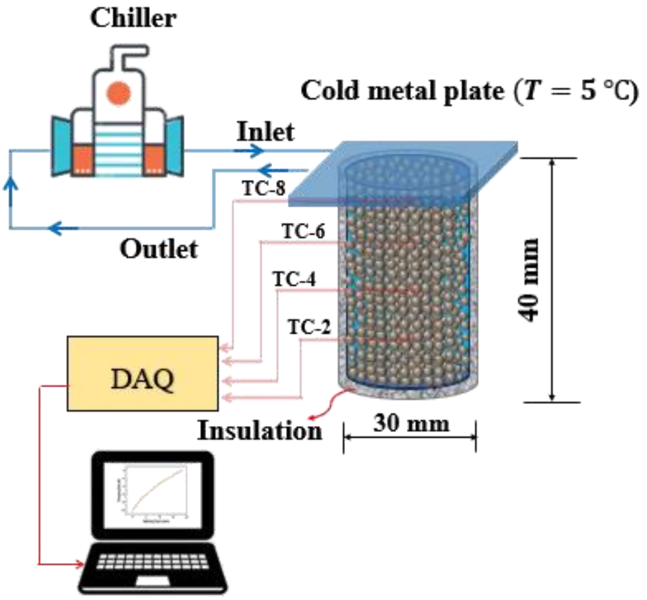

Fig. S1. Schematic of thermocouple-embedded experimental setup for studying melting process in vertically orientated porous samples.

S2 Phase Change Heat Transfer Analysis

To further understand the heat transfer mechanism during ice melting in porous media, a one-dimensional analysis for the phase change process is conducted by considering temperature change in the radial direction only without heat generation. As shown by the radial cross-section of porous media in Fig. 5(c) & 5(d) in the main manuscript, the annular region contains water in porous matrix with an effective thermal conductivity of $k_{water,eff}$, while icing porous media remains around the center with an effective thermal conductivity of $k_{ice,eff}$ as defined below:

$$k_{water,eff} = (1-\phi)k_{porous\ \mathrm{media}} + \phi k_{water} \tag{S1}$$

$$k_{ice,eff} = (1-\phi)k_{porous\ \mathrm{media}} + \phi k_{ice} \tag{S2}$$

where ϕ is the porosity of the porous media. A sharp interface between solid and liquid phases is assumed with temperature of the ice is close to its melting temperature. During the melting process, both sensible and latent heat transfer takes place. Stefan number, which is the ratio of sensible to latent heat, is defined as [1]:

$$S_t = \frac{c_p(T_s - T_f)}{\Delta h_f} \tag{S3}$$

where cp is the specific heat capacity of water, T_s is surface temperature of the NMR core, T_f is melting or fusion temperature of ice and Δh_f is the latent heat of fusion. For T_s = 5 °C, $c_p = 4180$ J/kg K, $\Delta h_f = 333.5$ KJ/kg and $T_f = 0$ °C, S_t is found to be 0.0627. As St < 0.1, the sensible heat transfer during the phase change process can be neglected [2]. With this assumption, the energy equations for ice and water regions (Fig. 5d in the main manuscript) are given by Eq. (S4) and Eq. (S5) respectively:

$$\frac{1}{r}\frac{d}{dr}\left(r\frac{dT_{ice}}{dr}\right) = 0,\ 0 < r < r_{mf} \tag{S4}$$

$$\frac{1}{r}\frac{d}{dr}\left(r\frac{dT_{water}}{dr}\right) = 0,\ r_{mf} < r < r_o \tag{S5}$$

where r_{mf} is the radial distance of melting front as marked in MRI images (Fig. 5c in the main manuscript). The corresponding boundary conditions are as follows: $\left.\frac{dT}{dr}\right|_{ice} = 0$ at $r = 0$, $T = T_f$ at $r = r_{mf}$ and $T = T_o$ at $r = r_o$. T_o represents surface temperature of the porous sample (Fig. 2c in the main manuscript). The ice-water interface energy transfer can be written as [1]:

$$\rho_{ice}\phi\Delta h_f\frac{dr_{mf}}{dt} = k_{water,eff}\left.\frac{dT_{water}}{dr}\right|_{r=r_{mf}} - k_{ice,eff}\left.\frac{dT_{ice}}{dr}\right|_{r=r_{mf}} \tag{S6}$$

By employing above-mentioned boundary conditions (i.e., $T = T_f$ at $r = r_{mf}$ and $T = T_o$ at $r = r_o$), Eq. (S4) can be written as:

$$\left.\frac{dT_{ice}}{dr}\right|_{r=r_{mf}} = 0 \tag{S7}$$

Similarly, Eq. (S5) can be transformed into Eq. (S8) while incorporating corresponding boundary conditions as:

$$\left.\frac{dT_{water}}{dr}\right|_{r=r_{mf}} = \frac{1}{r_{mf}} \frac{T_o - T_f}{\left(\ln r_o - \ln r_{mf}\right)} \qquad \text{(S8)}$$

Substituting Eqs. (S7-S8) into Eq. (S6):

$$\rho_{ice}\phi\Delta h_f \frac{dr_{mf}}{dt} = k_{water,eff} \frac{1}{r_{mf}} \frac{T_o - T_f}{\left(\ln r_o - \ln r_{mf}\right)} \qquad \text{(S9)}$$

By evaluating the variation of r_{mf} as in Fig. 5d in the main manuscript, Eq. (S9) can be used to estimate the surface temperature T_o, which increases slightly (within 3 degrees) during the whole melting process of porous media with 100-200 μm glass beads. This slight increase in temperature will reduce temperature difference between the porous sample and surrounding, eventually reducing melting rate in second stage of melting process ($t > 28$ min) as shown in Fig. 4d in the main manuscript.

S3 Signal amplitude vs dimensionless radial position of the melting front

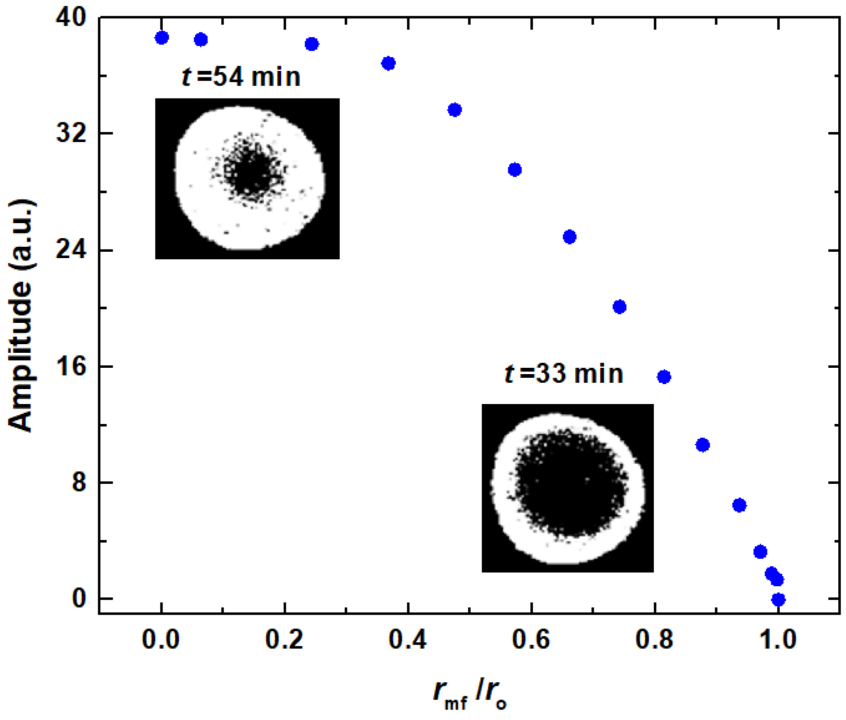

Fig. S2. T_2 Signal amplitude vs dimensionless radial position of the melting front (r_{mf} / r_o).

The MRI images were processed in MATLAB by modifying the function/script file available at MathWorks File Exchange [3].

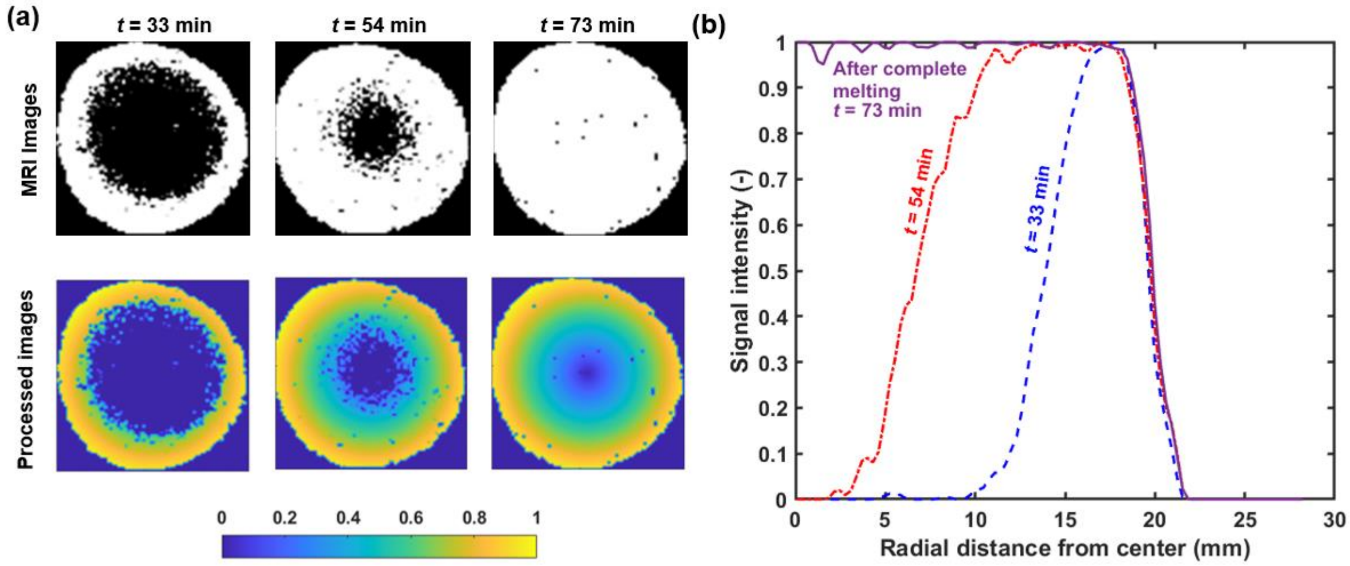

Fig. S3. Processing of MRI images in MATLAB for t =33 min, t =54 min and t =73 min (i.e., after complete ice melting). (b) Normalized signal intensity as a function of radial distance from the sample centre for different melting time.

S4 Characterization of Gravity Effect on Ice Melting by Temperature Distribution Measurements

Temperature distribution measurements for vertically oriented samples have been obtained, where the samples are slowly heated by a top surface at 5 ºC while the side wall and bottom surface areas are thermally insulated. Temperature changes of porous media with different glass bead sizes and orientations are summarized in Table S2. Fig. S4a shows the representative temperature reading for ice melting in porous media with the glass beads of 1-50 μm under vertical orientation. It is shown that the melting front took 986 seconds to reach the top thermocouple TC-8, while it took 1024 and 1102 seconds to reach thermocouple TC-6, and TC-2, respectively. Fig. S4b shows the effect of pore size and sample orientation on time taken by the melting front to reach TC-2. The results clearly indicate that melting time increases with the increase in pore size for horizontally oriented samples. However, an opposite trend is observed for vertically orientated samples as increase in pore size reduces melting time. As shown in Table S2 and Fig. S4b, the results under vertical orientation indicate faster ice melting in large glass beads as compared to small glass beads. This means that the melting front was moving faster inside large pores than small pores, which shows that natural convection is the major heat transfer mode in the vertically oriented porous media. Along with the melting ice within the top part of the sample, the density increases because of water density inversion behavior, and gravity drives water to the bottom section to displace the colder ice. This happens at a relatively fast rate among the large-sized pores, because water has lower flow resistance in larger pores and natural convection enhances heat transfer besides conduction.

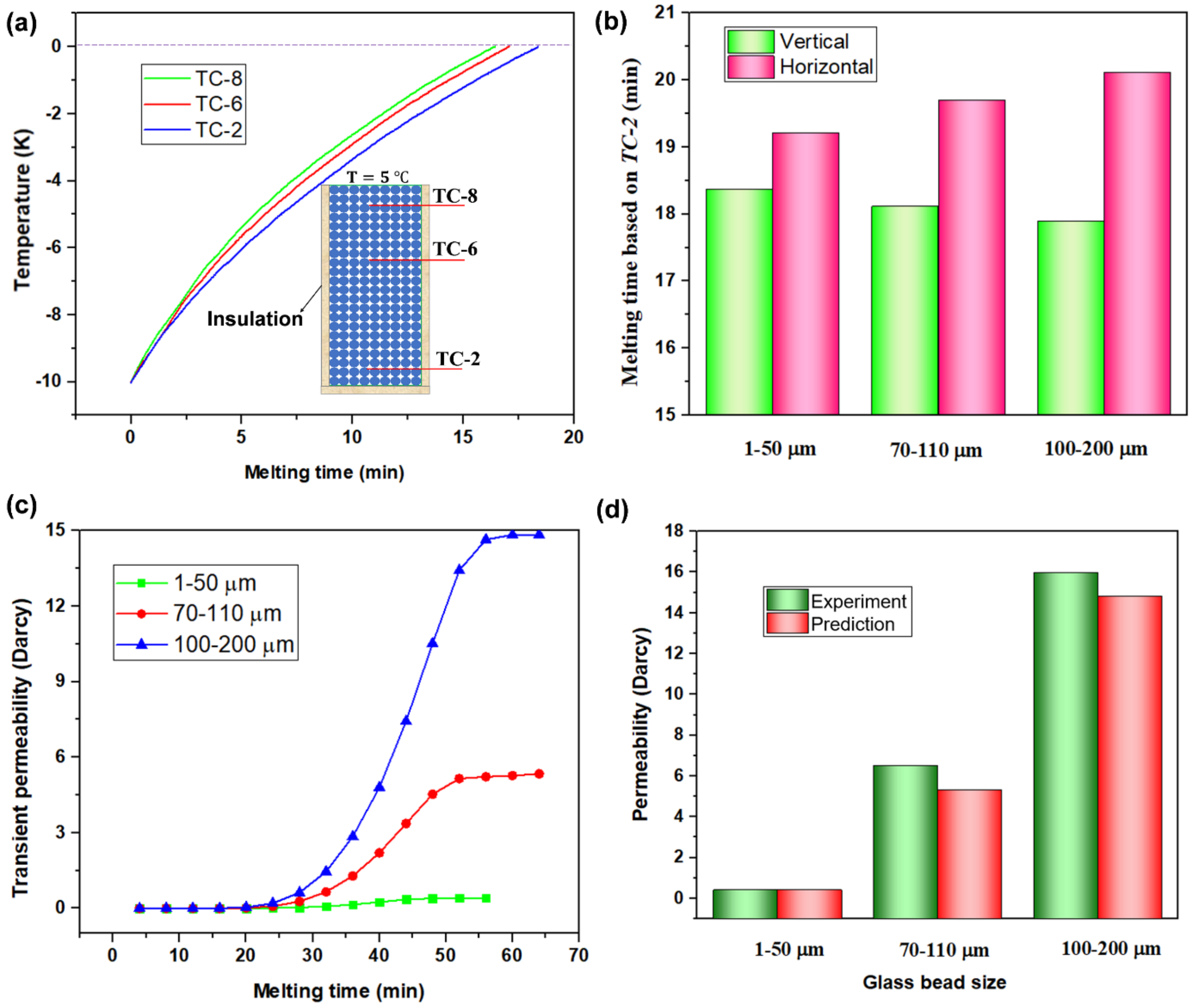

Fig. S4. (a) Transient temperature distribution during ice melting in the tight porous medium with 1-50 μm glass beads; (b) Effect of glass beads size on time taken by the melting front to reach thermocouple TC-2. Permeability of porous media made of different-sized glass beads; (c) Transient permeability calculated using Carmen-Kozeny equation; (d) Comparison of experimentally measured and predicted permeability at full saturation.

As a measure of the fluid flow velocity across porous media, permeability has been recognized as the major factor that causes faster melting among large glass beads in vertical orientation [4]. For rocks, natural soil, and other heterogeneous porous media, permeability behaves anisotropically and exhibits different values along different directions. However, for homogenous and isotropic porous media, permeability is the same in all directions. The theoretical permeability is measured with the Carman Kozeny equation [5,6] while the falling head permeability method [7] is used for experimental permeability.

$$K = \frac{d_m^2 \gamma^3}{175(1-\gamma)^2} \tag{S10}$$

where d_m is the pore diameter, and γ is the liquid fraction. Larger pore size and liquid fraction usually result in higher permeability. Although Eq. (S10) is an empirical correlation, it gives good results for a porous medium that contains particles of spherical shape with a narrow size range. The equation is best suited to measure permeability during the phase change process as it can provide the permeability values throughout the melting process as a function of the liquid fraction. The value of the liquid fraction is zero at the beginning when it is in ice state, and then it increases to 1 when it is fully saturated with water. Therefore, the permeability must be initially zero, and then it

keeps increasing. In porous media with large glass beads and pores, gravity has a strong impact and imposes high permeability.

The transient permeability is predicted throughout the melting process by using the Carman–Kozeny equation, as seen in Fig. S4c. At the start, the liquid fraction is very small, and permeability is also small for all the porous media. This is due to the exponent of the liquid fraction, as it has a dominant effect in reducing the permeability. However, after t = 25 minutes it rises exponentially for higher size glass beads. It can be seen that the permeability is proportional to the pore size. For the tight porous media with the smallest bead size (1-50 μm), the permeability reaches a maximum value of 0.41 Darcy, which shows the strong resistance of porous media for fluid flow. In addition, permeability is experimentally measured at a fully saturated state and compared with the predicted maximum permeability, where the maximum permeability refers to full saturation when the liquid fraction is 1. The comparison of results given in Fig. S4d shows that predicted values for permeability are in good agreement with the experiments. For instance, the measured permeability is 15.98 Darcy for glass beads with a size range of 100-200 µm while the predicted value is 14.83 Darcy. The permeability of loose porous media with large glass beads is higher than the permeability of tight porous media, which could be a reason for the higher melting rate in the large glass beads than the small glass beads under the vertical orientation. In general, it does show the significant impact of permeability in controlling and facilitating the movement of the ice-melting front in porous media.

Table S1: Estimation of melting point depression (ΔT_m) of water confined in pore and throat of porous media based on NMR data

**Glass beads size (µm)**	ΔT_{pore} (°C)	ΔT_{throat} (°C)
1-50	0.006	0.06
70-110	0.002	0.02
100-200	0.001	0.01

Table S2: Melting time of various icing porous media under vertical and horizontal orientations at locations TC-2 to TC-8

**Melting time of homogeneous porous media under vertical orientation**			
**Glass beads size (µm)**	TC-2 (sec)	TC-6 (sec)	TC-8 (sec)
1-50	1102	1024	986
70-110	1087	1013	980
100-200	1074	1002	969
**Melting time of homogeneous porous media under horizontal orientation**			
**Glass beads size (µm)**	TC-2 (sec)	TC-6 (sec)	TC-8 (sec)
1-50	1153	1101	1083
70-110	1182	1131	1105
100-200	1207	1158	1140
**Melting time of heterogeneous lunar highlands simulant under horizontal orientation**			
**Particle size (µm)**	TC-2 (sec)	TC-6 (sec)	TC-8 (sec)
0.01-1000	1198	1153	1137

References

1 Weaver, J. A. & Viskanta, R. Melting of frozen, porous media contained in a horizontal or a vertical, cylindrical capsule. *International Journal of Heat and Mass Transfer* **29**, 1943-1951, doi:https://doi.org/10.1016/0017-9310(86)90013-X (1986).

2 Bauer, T., Steinmann, W.-D., Laing, D. & Tamme, R. Thermal energy storage materials and systems. *Annual Review of Heat Transfer* **15** (2012).

3 Sergey Loginov (2024). Very Fast Radial Profile (https://www.mathworks.com/matlabcentral/fileexchange/101480-very-fast-radial-profile), MATLAB Central File Exchange. Retrieved January 28, 2024.

4 Alfarisi, O., Ouzzane, D., Sassi, M. & Zhang, T. The Understanding of Intertwined Physics: Discovering Capillary Pressure and Permeability Co-Determination. *arXiv preprint arXiv:2112.12784* (2021).

5 Nield, D. *Convection in Porous Media, Fifth Edition*. (2017).

6 Babchin, A. J., Bentsen, R., Faybishenko, B. & Geilikman, M. B. On the capillary pressure function in porous media based on relative permeabilities of two immiscible fluids: Application of capillary bundle models and validation using experimental data. *Advances in Colloid and Interface Science* **233**, 176-185, doi:https://doi.org/10.1016/j.cis.2015.07.001 (2016).

7 Zhang, Y., Li, H., Abdelhady, A. & Yang, J. Comparative laboratory measurement of pervious concrete permeability using constant-head and falling-head permeameter methods. *Construction and Building Materials* **263**, 120614, doi:https://doi.org/10.1016/j.conbuildmat.2020.120614 (2020).
